# Missingness in Eligibility Criteria for Target Trial Emulation in EHR With Survival Outcomes

**DOI:** 10.1002/sim.70500

**Published:** 2026-04-07

**Authors:** Jenny Shen, Kristin A. Linn, Amy S. Clark, Ronac Mamtani, Rebecca A. Hubbard

**Affiliations:** ^1^ Department of Biostatistics, Epidemiology, and Informatics, Perelman School of Medicine University of Pennsylvania Philadelphia Pennsylvania USA; ^2^ Abramson Cancer Center Hospital of the University of Pennsylvania Philadelphia Pennsylvania USA; ^3^ Department of Biostatistics Brown University School of Public Health Providence Rhode Island USA

**Keywords:** breast cancer, electronic health records, missing data, multiple imputation

## Abstract

In certain settings, when conducting a randomized trial would be infeasible, electronic health records (EHR) can be used to emulate a target trial and estimate causal effects of an intervention. This process involves specifying the elements of a hypothetical trial protocol and applying these to the design of an observational study conducted with EHR data (or other observational data source). One element of target trial specification includes defining eligibility criteria. However, defining the eligible population with EHR can be complicated by missingness in eligibility‐defining variables. Multiple imputation (MI) is one common approach to missingness in EHR data, but it is unclear whether imputation of eligibility criteria should occur before or after excluding ineligible individuals. Motivated by a target trial emulation of two treatments for advanced breast cancer, we explore this question when estimating the average causal effect under a target trial framework with survival outcomes. We illustrate how alternative MI strategies perform using simulated data and in a real‐world analysis of oncology EHR data. We found that in most settings with high proportions of missingness in eligibility‐defining variables, imputing missing data using a flexible imputation model, such as a random forest, prior to excluding ineligible individuals resulted in lower bias than complete case analysis or imputation after excluding ineligible individuals. Choices about how to handle practical challenges such as this in the application of target trial emulation to messy, real‐world data sources can have substantial effects on causal parameter estimation and should be carefully considered to ensure that the results of observational studies are as rigorous as possible.

## 1 Introduction

Using electronic health records (EHR) to study comparative effectiveness and safety of competing treatment strategies or support clinical decision‐making in the absence of clinical trial data has increasingly gained traction [1, 2]. In scenarios in which conducting a randomized trial would be infeasible due to ethical or logistical constraints, under certain assumptions, EHR can be leveraged for causal inference. One causal inference approach, the target trial emulation paradigm [3], involves specifying the randomized trial that would ideally have been conducted and aligning an observational study design with that target trial. A number of study design elements must be specified for the trial emulation, including eligibility criteria, treatment strategies, and causal contrasts of interest [4]. Ultimately, target trial emulations seek to address biases common in observational studies, such as selection bias and immortal time bias [3, 5].

The study sample should be selected from the population of individuals captured in the relevant real‐world database by applying study‐specific inclusion and exclusion criteria. However, there are several challenges to characterizing inclusion/exclusion criteria in EHR data. Some clinical trial inclusion criteria are not documented in the EHR at all. One study on clinical trial eligibility criteria for cancer treatments highlighted that many eligibility criteria used in cancer trials are not captured in EHR data [6]. Another common challenge in EHR data is ambiguity between a lack of information on a characteristic and the absence of the characteristic. For example, when a specific diagnosis code is not found in a patient's record, it is unknown whether the patient was not assessed for that condition or the patient was assessed and does not have the condition of interest. In the current study, we consider the common case where information about a data element, such as a biomarker, is captured for some individuals and explicitly absent for others. How this type of missingness in eligibility criteria is addressed is important because outcomes and treatment effectiveness may vary for eligible and ineligible patients [7]. One common approach is to exclude patients with missing data [8, 9], but simply excluding individuals with missing eligibility criteria could lead to selection bias. Existing literature has explored handling missingness in variables pertinent to analyses (e.g., exposures, outcomes, and confounders), but limited work exists investigating methods for addressing missingness in inclusion criteria [10, 11, 12, 13]. Practices for handling missing data on eligibility criteria are inconsistent across target trial emulations and not well‐documented. Presently, it is unclear how to best address bias in target trial emulation resulting from missingness in inclusion/exclusion criteria.

A few prior studies have investigated multiple imputation (MI) for missing eligibility criteria. Tompsett et al. [12] used MI to recover missing eligibility data when estimating the average causal effect (ACE) of a point exposure. Austin et al. [13] focused on a scenario where a variable was both eligibility‐defining and the exposure of interest, and the authors compared different MI strategies for addressing missingness. Another recent study examining the issue of missingness in eligibility criteria focused on time‐to‐event endpoints but considered a series of target trials that are conducted periodically (e.g., annually). Under this framework, eligibility criteria can be assessed at multiple points in time. The authors proposed fully parametric methods focusing on the estimation of a per‐protocol effect or an intention‐to‐treat effect [14]. In considering a sequence of target trials, multiple imputation was intractable for handling missingness. The authors instead used inverse probability weighting to address missingness [14].

A key analytic decision is whether to impute missing data before or after applying eligibility criteria. This decision has implications for bias, precision, and accurate standard error estimation. Two common MI strategies are to exclude‐then‐impute or impute‐then‐exclude. Exclude‐then‐impute consists of excluding ineligible individuals based on non‐missing values, followed by imputing missing data in the remaining subset. Impute‐then‐exclude consists of imputing missing data followed by excluding ineligible individuals. Under the impute‐then‐exclude approach, Giganti and Shepherd [15] investigated variance estimation for MI and found that Rubin's rules lead to incorrect variance estimation. Although some target trial emulations have implemented exclude‐then‐impute for missingness [16, 17, 18], prior work suggests that impute‐then‐exclude may reduce bias [12, 13].

In this study, we explored whether impute‐then‐exclude could lead to reduced bias in estimation of the ACE for target trial emulation with survival outcomes and whether the use of machine learning for imputation models could improve performance relative to traditional, parametric imputation. Previous work for handling missingness in target trial emulation has focused on continuous or binary outcomes. This current study further extends prior work by focusing on estimation of a causal estimand and conducting a head‐to‐head comparison of impute‐then‐exclude and exclude‐then‐impute [12].

Our work was motivated by a randomized clinical trial of two competing treatment strategies for metastatic breast cancer (PARSIFAL), Palbociclib in combination with either Fulvestrant or Letrozole [19]. Results from this trial found that the two treatment combinations performed comparably, reporting a hazard ratio of 1.13 (95% CI of 0.89–1.45, *p* = 0.32) for progression‐free survival. The results of this trial were recently emulated in an observational study that compared overall survival for patients receiving the two treatments [20]. This target trial emulation estimated an overall survival hazard ratio of 1.07 (95% CI of 0.86–1.35). In the previously published target trial emulation that used EHR data from the US Oncology Network, the authors addressed missingness in eligibility criteria using an exclude‐then‐impute strategy. To investigate the performance of this missing data strategy compared to alternatives, we used data from the nationwide Flatiron Health electronic health record (EHR)‐derived de‐identified database to conduct a trial emulation and compare different MI strategies for missingness in eligibility criteria.

Our work is organized as follows. First, we discuss the estimation of the ACE with survival outcomes and provide a brief overview of methods when eligibility criteria are subject to missingness. We then compare the performance of MI strategies for missingness in eligibility criteria when estimating causal effects of survival outcomes in target trial emulations with parametric and plasmode simulations. In Section 4, we conduct a target trial emulation with the real‐world oncology EHR data to compare treatments for metastatic breast cancer.

## 2 Methods

### 2.1 Estimation of the ACE

We investigated estimation of the ACE in settings with survival outcomes using a target trial emulation framework. We emulated the PARSIFAL trial [19] using real‐world data from the longitudinal, Flatiron Health EHR‐derived de‐identified database containing patient‐level structured and unstructured data, curated via technology‐enabled abstraction. During the study period, the de‐identified data originated from approximately 280 cancer clinics (∼800 sites of care) across the US. Data from Flatiron Health included information for demographics, progression‐free survival, clinical history, and other patient attributes [21, 22]. The majority of patients in the database originate from community oncology settings. Patients with a birth year of 1938 or earlier may have an adjusted birth year in Flatiron datasets due to patient de‐identification requirements. Adult patients with stage IV breast cancer during 2011–2023 who initiated first‐line treatment for metastatic breast cancer with one of the two treatments of interest from April 13, 2015, to November 1, 2023, were included in the Flatiron Health cohort.

Under the target trial emulation framework, we are interested in estimating the average causal effect (ACE) of a treatment A∈{0,1} on survival among individuals who meet the eligibility criteria. To define this eligible population in the real‐world cohort, we adopt inclusion and exclusion criteria similar to those used in the PARSIFAL trial [19] and a related trial emulation [20]. To be included, individuals must have initiated first‐line therapy (in the metastatic setting) with Fulvestrant and Palbociclib or Letrozole and Palbociclib; were 18 years or older at the index date (i.e., start date of first‐line therapy); and had no systemic anti‐cancer therapy following diagnosis of metastatic disease and prior to the index date. Individuals were excluded if they had confirmed diagnosis with estrogen receptor (ER)‐negative disease; confirmed diagnosis with human epidermal growth factor receptor 2 (HER‐2) positive disease; record of central nervous system (CNS) metastases prior to index date; prior treatment with Letrozole or Anastrozole ≤ 12 months from index date; prior treatment with any cyclin‐dependent kinase (CDK) 4/6 inhibitor (Ribociclib, Palbociclib, Abemaciclib) ≤ 12 months from index date; were male; or had Eastern Cooperative Oncology Group (ECOG) performance status scores greater than 2. These design elements, as well as additional eligibility criteria for the PARSIFAL trial, are shown in Table 1.

**TABLE 1:** Description of eligibility criteria for the PARSIFAL randomized trial and target trial emulation using real‐world data.

Type of eligibility criteria	PARSIFAL trial eligibility criteria	Target trial emulation using Flatiron
**Inclusion**		
	Age at least 18 years with locally advanced breast cancer not amenable to curative therapy	Age at least 18 years with diagnosis of breast cancer
		Initiated first‐line therapy (metastatic setting) of “Letrozole, Palbociclib” or “Fulvestrant, Palbociclib”
	No prior chemotherapy in the metastatic aetting	No systemic anti‐cancer therapy following initial record indicating metastatic disease and prior to index date
	Confirmed ER+ histology	No records indicating ER‐negative histology prior to or on index date
	Adequate organ and marrow function, resolution of all toxic effects of prior therapy or surgical procedures	
	Measurable disease as per RECIST v1.1 or non‐measurable disease	
	Postmenopausal status	
	Resolution of all acute toxic effects of prior anti‐cancer therapy or surgical procedures to NCI‐CTCAE version 4.0 grade equal to or minor than 1	
**Exclusion**		
	Confirmed HER‐2+ Disease	Confirmed diagnosis with HER‐2+ disease
	Known uncontrolled or symptomatic CNS metastases	Record of CNS metastases prior to index date
	Prior (neo)adjuvant endocrine therapy with DFI ≤12 months from completion of treatment	Treatment with Letrozole or anastrozole ≤12 months from index date
		Prior treatment with any CDK 4/6 inhibitor (Ribociclib, Palbociclib, Abemaciclib) ≤12 months from index date
	ECOG > 2	ECOG > 2
		Received Palbociclib without Letrozole or Fulvestrant
		Male gender
	Major surgery within 4 weeks of start of study drug	
	Patients with an active, bleeding diathesis	
	Serious concomitant systemic disorder incompatible with the study	
	Known hypersensitivity to Letrozole, Fulvestrant, or any of their excipients, or to any Palbociclib excipients	
	Are unable to swallow tablets	
	QTc > 480 msec on basal assessments, personal history of long or short QT syndrome, Brugada syndrome, or known history of QTc prolongation, or Torsade de Pointes	
	Uncontrolled electrolyte disorders that can compound the effects of a QTc‐prolonging drug	
	Patients with rapidly progressive visceral disease or visceral crisis	
	Locally advanced breast cancer candidate for a radical treatment	
	Chronic daily treatment with corticosteroids with a dose of at least 10 mg/day methyl prednisolone equivalent	

Let $Z\in {\mathbb{R}}^{q}$ represent the q eligibility criteria variables, and let $I_{Z}$ be an indicator variable for whether an individual met the eligibility criteria, with $I_{Z}=1$ representing an eligible individual. $R_{Z}$ is an indicator of completeness for the eligibility criteria, with $R_{Z}=1$ representing no missingness for the eligibility criteria. T denotes the time until the occurrence of an event and $X\in {\mathbb{R}}^{p}$ denote a vector of p baseline covariates. Let $T^{a}$ represent the potential survival time that would have been observed if a subject had received treatment A=a.

Let the counterfactual survival probability of an eligible individual at time t given treatment assignment A be denoted by $P(T^{A}>t|I_{Z}=1)$ [23, 24]. We define the ACE among the eligible population to be the difference in survival probabilities at time t:

$$ACE(t)=P(T^{1}>t|I_{Z}=1)-P(T^{0}>t|I_{Z}=1).$$

To identify ACE(t), we make the following assumptions, conditional on $I_{Z}=1$:

1. No interference: Counterfactual survival times are independent of the treatment assignment of other individuals [25].
2. Counterfactual consistency: $P(T^{a}=t|A=a)=P(T=t|A=a)$ such that we observe the potential survival time that corresponds to the treatment A=a received, regardless of how A was assigned.
3. Conditional exchangeability: $T^{a}\;⊥\;A|X,Z$.
4. Positivity: P(A=a|X,Z)>0 for all X,Z [24].
5. Noninformative censoring: $T^{A}\;⊥\;C|A,X,Z$, where C represents censoring time.

Under these assumptions, we have the following result [23, 24]:

$$\begin{array}{cc} & P(T^{a}>t|I_{Z}=1,X=x_{i},Z=z_{i})\\ & \;=S(t|I_{Z}=1,A=a,X=x_{i},Z=z_{i}), \end{array}$$

where S(t)=P(T>t). The conditional ACE can then be identified as the difference in the conditional survival probabilities between treatment groups at time t:

$$\begin{array}{cc} ACE(t) & =S(t|I_{Z}=1,A=1,X=x_{i},Z=z_{i})\\ & \;-S(t|I_{Z}=1,A=0,X=x_{i},Z=z_{i}). \end{array}$$

To obtain the unconditional ACE, the target estimand, we marginalize over the distribution of X and Z.

In our simulations and real data analysis, we estimated the unconditional ACE with a model‐based approach using a Cox proportional hazards model with estimated survival probabilities adjusted via direct standardization (i.e., G‐computation) [24, 26]. After estimating the model, we made predictions for the survival probability at time t for all individuals in the data set under each of the two possible treatments and averaged the predictions. We then estimated the unconditional ACE by taking the differences in those means. We have further assumed that the Cox model is correctly specified to produce unbiased estimates of the conditional survival probabilities.

The unconditional ACE can be estimated as

(1) $$\begin{array}{ll} & \;Ŝ(t|I_{Z}=1,A=1)-Ŝ(t|I_{Z}=1,A=0)=\\ & \;\frac{1}{n}\overset{n}{\underset{i=1}{\sum }}Ŝ(t|I_{Z}=1,A=1,X=x_{i},Z=z_{i})\\ & \;-\frac{1}{n}\overset{n}{\underset{i=1}{\sum }}Ŝ(t|I_{Z}=1,A=0,X=x_{i},Z=z_{i}) \end{array}$$

In practice, eligibility criteria may be subject to missingness. In that case, we may take a complete case approach in which the conditional ACE is approximated from individuals with complete eligibility information ($I_{Z}=1,R_{Z}=1$), where we marginalize estimates of the conditional ACE for the eligible population without missing data:

$$\begin{array}{cc} & Ŝ(t|I_{Z}=1,R_{Z}=1,A=1,X=x_{i},Z=z_{i})\\ & \;-Ŝ(t|I_{Z}=1,R_{Z}=1,A=0,X=x_{i},Z=z_{i}). \end{array}$$

Under the complete case approach, however, including only individuals with observed eligibility criteria could induce selection bias, potentially leading to biased estimates of the conditional ACE.

Put another way, for the complete case approach, the distribution of confounders in the eligible population with no missing data may not match the distribution of confounders for the full eligible population. Marginalizing over the complete case population may therefore lead to a distorted estimate of the unconditional ACE. In lieu of conditioning only on individuals with observed eligibility criteria, which could lead to subsequent selection bias, we could consider multiple imputation for handling missingness in eligibility criteria.

As stated in Tompsett et al. [12], multiple imputation can be used to recreate the joint distribution of the treatment, outcome, and confounders of the full eligible population ($I_{Z}=1$) among the complete eligible population ($I_{Z}=1$ and $R_{Z}=1$). Multiple imputation is able to recover this joint distribution through leveraging observed data to reconstruct missing values [27].

### 2.2 Methods for Missingness in Eligibility Variables

We explored four different methods for data subject to missingness in eligibility criteria: A complete case analysis and three MI strategies. In the complete case analysis, we excluded individuals with missingness before further restricting to individuals who met the eligibility criteria. We then proceeded with estimating the unconditional ACE. For the MI strategies, we considered exclude‐then‐impute with parametric multiple imputation via chained equations (MICE), impute‐then‐exclude with parametric MICE, and impute‐then‐exclude with MICE using random forest, a machine learning approach. Random forests can more flexibly model relationships among variables, which may be particularly helpful with complex, real‐world EHR data. Including this imputation strategy allowed us to explore whether we could improve the congeniality of the imputation and outcome models, a limitation of excluding after imputation, by using a random forest to impute missing eligibility criteria. Non‐congeniality arises when the imputation model and the analysis model have differing assumptions. In the case of impute‐then‐exclude, individuals included in the imputation model may be excluded from the analysis model. The associated assumptions about the joint distribution among outcomes, treatment variable, and confounders for individuals used for the imputation model and those used for the analysis model can be discordant. This discordance between imputation and analysis models results in non‐congeniality for impute‐then‐exclude [15].

In all three imputation strategies, we used MICE [28] and imputed separately for each treatment level to account for potential heterogeneity in treatment effects [12]. For imputing continuous, binary, and categorical variables under parametric MICE, we used predictive mean matching, logistic regression, and polytomous regression, respectively. For imputation using random forests, the imputation was carried out by fitting a random forest. Under the random forest approach, a set of classification trees was fit on a bootstrapped sample of the data, and each imputed value was chosen as the prediction of a randomly chosen tree. We implemented random forest imputation using the default random forest settings from the CALIBERrfimpute R package, which included fitting ten classification trees with the number of nodes determined by the number of observations in the data [29].

For exclude‐then‐impute, we assumed that not all exclusion criteria may be measured on individuals. For those with observed information for eligibility‐defining variables, we proceeded with excluding subjects that failed to meet the eligibility criteria. For the resultant sample of individuals, which consisted of individuals who met the eligibility criteria or had missing values for the eligibility variable, we used MI to impute missing values of the eligibility variable. Consider, for instance, a case where an individual had missingness in all eligibility variables except age. If their age happened to be ≥ 18, thereby satisfying the criteria to be included, then this individual would be retained in the analytic sample. Eligibility variables with missingness for this individual would then be imputed. However, if their age happened to be < 18, this individual would be excluded. As recommended in the literature for imputing in survival settings, the imputation model included the treatment variable, other relevant covariates, the event status indicator, and the estimated baseline cumulative hazard, estimated using the Nelson–Aalen estimator [30]. We imputed missing values M times, obtaining M imputated data sets. In each imputed data set, we estimated the unconditional ACE at a pre‐selected time t. We obtained the final estimate of the ACE by averaging estimates of the ACE across the M imputed data sets.

For impute‐then‐exclude, we first used MI to impute missingness in eligibility‐defining variables. We used the same imputation method as used under the exclude‐then‐impute approach and imputed M times. The same set of covariates was included, and the same imputation procedures were followed. In each imputed data set, we then excluded subjects that failed to meet the eligibility criteria before estimating the ACE.

For all approaches, confidence intervals (CI) were constructed using percentiles of the bootstrap distribution. The Rubin's Rules MI variance estimator has been demonstrated to be biased due to incompatibility between the imputation and analysis models for the impute‐then‐exclude setting [15]. Thus, a bootstrap approach was used to obtain correct standard errors. To construct the bootstrap CI, we followed the approach of Schomaker et al. [31]. We first obtained B bootstrap samples of the data. We then implemented one of the aforementioned methods for handling missingness for each of the B data sets to obtain B estimates of the unconditional ACE. Upon ordering the bootstrap estimates, we derived a percentile‐based bootstrap confidence interval. Single imputation (M=1) nested within bootstraps has been shown to be adequate in these settings while reducing computational burden [12, 31, 32, 33]. We therefore set M=1 in numerical studies presented below.

## 3 Simulation Studies

We conducted parametric and plasmode simulation studies to compare the performance of MI strategies for addressing missingness in eligibility criteria when targeting estimation of the ACE with survival outcomes. Plasmode simulations used EHR data comparing treatments for advanced breast cancer to explore the performance of alternative approaches in the setting of a real‐world target trial emulation. We provide results using the full sample (i.e., no missingness) and using data subject to missingness under the complete case, exclude‐then‐impute, and impute‐then‐exclude approaches. We also obtained results with varying proportions of missingness for the eligibility‐defining variable.

### 3.1 Parametric Simulations

We first simulated data for a super‐population of size $n=10^{6}$. This superpopulation was used to obtain benchmark estimates for the true value of the ACE. We let Z denote a 3‐level categorical variable representing an inclusion/exclusion criterion taking a value of 1, 2, or 3. Similar to simulations in Austin et al. [13], Z=3 was defined to be an exclusion criterion, and subjects with Z=3 were excluded from analysis. We generated Z from a multinomial distribution using the following probabilities: P(Z=1)=P(Z=2)=0.35 and P(Z=3)=0.3. X was a continuous baseline covariate with a mean that varied across levels of Z, where X∼N(−0.4+0.2∗Z,1). The binary treatment A was simulated using a logistic model:

$$\begin{array}{cc} & \;logit(P(A=1|Z,X))=\beta_{A0}+\beta_{A1}I(Z=2)+\beta_{A2}I(Z=3)\\ & \;+\beta_{A3}X+\beta_{A4}I(Z=3)X. \end{array}$$

The true values of the coefficients were $\beta_{A0}=0.40,\beta_{A1}=log(0.90),\beta_{A2}=log(0.85),\beta_{A3}=log(1.9)$ and $\beta_{A4}=log(1.5).$ For simulating survival times T, let $\lambda_{0}$ represent the baseline hazard. Event times were simulated from the model T∼Exp(λ), where

$$\begin{array}{cc} \lambda & =\lambda_{0}exp(\beta_{1}I(Z=2)+\beta_{2}I(Z=3)+\beta_{3}X+\beta_{4}I(Z=3)X\\ & \;+\beta_{5}A+\beta_{6}I(Z=3)A)). \end{array}$$

Here, $\lambda_{0}=0.001$, $\beta_{1}=log(1.75),\beta_{2}=log(1.75),\beta_{3}=log(0.25),\beta_{4}=log(0.5),\beta_{5}=log(0.5)$, and $\beta_{6}=log(0.9)$. The magnitude of the coefficients was selected to approximate the effect sizes used in Austin et al. [13]. Censoring times were generated as C∼Exp(θ), where θ was chosen to achieve a target proportion of censoring of 0.2, similar to the censoring proportion in the real‐world breast cancer example. The observed time for an individual was the minimum of the event time and censoring time, and an event indicator was defined as δ=I(T≤C). Let Ĥ represent the Nelson–Aalen estimator for the cumulative hazard.

To simulate missing at random (MAR) missingness, we specified a logistic model based on X,A,δ, and a function of the cumulative hazards, $H^{*}=\frac{Ĥ}{sd(Ĥ)}$. To simulate missingness, we used a logistic model for the probability of missingness:

$$\pi_{i}=logit(P(R_{Z}=0))=\alpha +\beta_{X}X_{i}+\beta_{\delta }\delta_{i}+\beta_{A}A_{i}+\beta_{H^{*}}H_{i}^{*}.$$

The intercept α was chosen to target a set proportion of missingness, which varied across simulations. The coefficients were set to the following values: $\beta_{X}=\beta_{\delta }=\beta_{A}=\beta_{H^{*}}=1$. In our parametric simulations, we investigated a range of missingness in Z from 10% to 60%.

Samples of size n=1000 or n=10000 were randomly sampled from the same super‐population for 1000 Monte Carlo simulation iterations per simulation scenario. An oracle estimate of the unconditional ACE(t) was obtained using the complete super‐population with t set to the median time to survival for the complete super‐population. This median time was also used for estimating the unconditional ACE(t) under each of the methods used for addressing missingness in Z. For each of the methods, we calculated the percent relative bias across the 1000 simulation replicates as

$$100\times \frac{\frac{1}{1000}(\sum_{k=1}^{1000}{\hat{ACE(t)}}_{k}-ACE(t))}{ACE(t)},$$

where ${\hat{ACE(t)}}_{k}$ is the estimate of the unconditional ACE(t) obtained in the kth simulation iteration. We also estimated the empirical coverage probability of estimated 95% confidence intervals using bootstrapping, the root mean squared error of ACE estimates (RMSE), and the Monte Carlo Error (MCE) for relative bias of estimates for the ACE. To estimate coverage probabilities, we used bootstrap confidence intervals with B=1000 bootstrap samples for each iteration.

**FIGURE 1:**
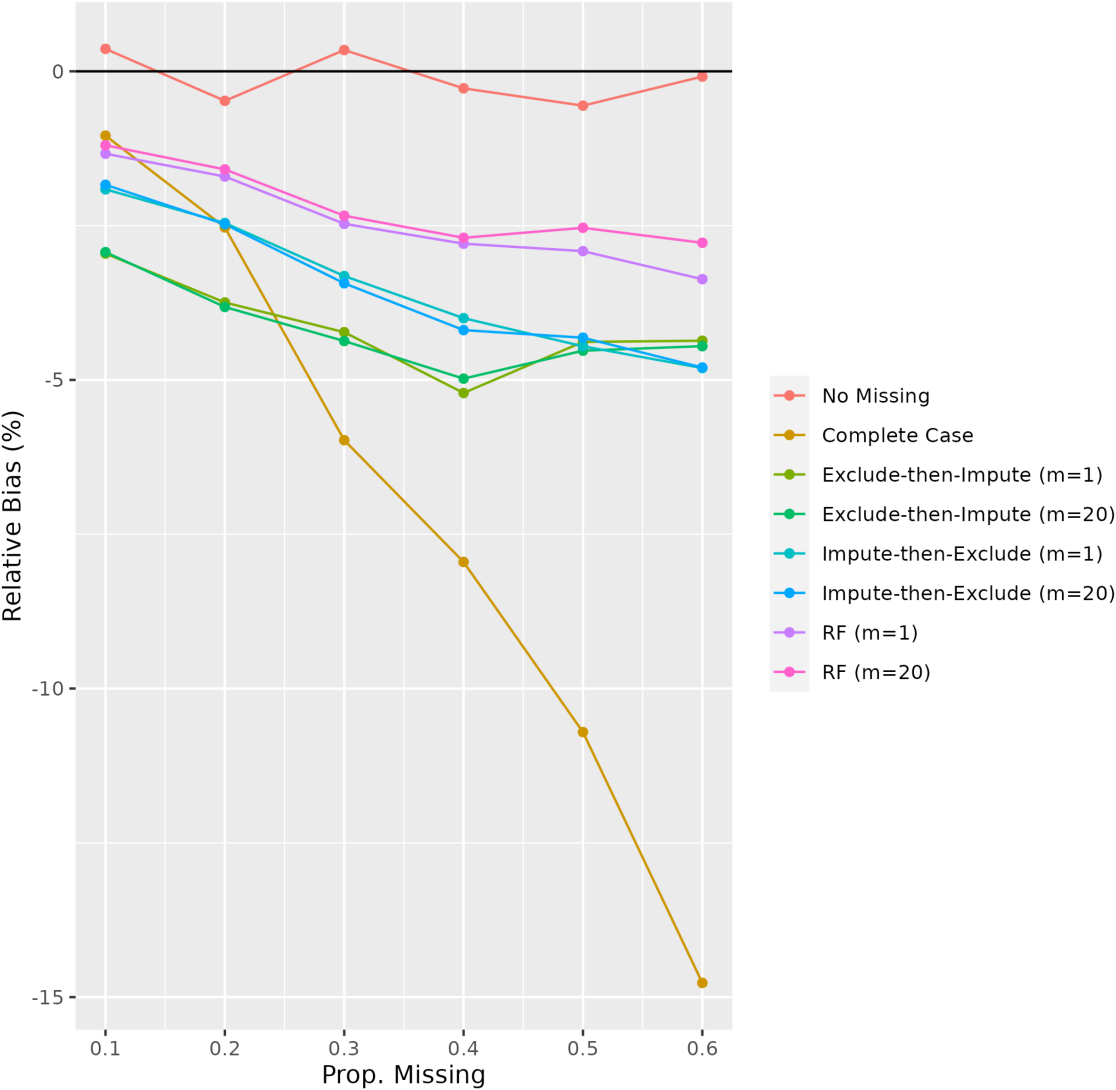
Relative bias of missing data strategies for proportion missingness in an eligibility‐defining variable ranging from 0.1 to 0.6 based on parametric simulations. Results are based on 1000 simulation iterations with sample sizes of n=1000. RF = random forest.

Estimates of the percent relative bias in the ACE for each of the methods across varying proportions of missingness for sample size n=1000 are shown in Figure 1. Estimates of the relative bias, coverage, RMSE, and MCE for all methods in the setting of 30% missingness in Z are provided in Table 2. The true unconditional ACE estimated from the complete super‐population using a median time of about 409 days was ACE=0.152. Similar results for the n=10000 setting are presented in the Supplement.

**TABLE 2:** Simulation results for relative bias, 95% confidence interval coverage probabilities, root mean squared error (RMSE), and Monte Carlo error (MCE) for relative bias of estimates of the average causal effect (ACE). Results are reported for different methods under 30% missingness in Z.

Method	Relative bias (%)	Coverage	RMSE	MCE for relative bias
No missing data	0.47	95.2	0.020	0.412
Complete case	−5.92	94.2	0.027	0.532
Exclude‐then‐Impute parametric	−4.46	94.5	0.020	0.382
Impute‐then‐Exclude parametric	−3.36	95.5	0.020	0.413
Impute‐then‐Exclude random forest	−2.12	97.1	0.020	0.415

As seen in Figure 1, the complete case analysis was highly biased, with bias increasing proportionally to the amount of missingness in eligibility criteria. Among the MI strategies, the performance of exclude‐then‐impute was generally the poorest, with a higher percent relative bias. The impute‐then‐exclude approaches maintained less than 5% relative bias across varying proportions of missingness, although performance declined with increasing amounts of missingness. Imputation using random forests had slightly less bias than parametric imputation. Results for the n=10000 setting were similar. However, in this larger sample size scenario, the three MI approaches performed more similarly, with exclude‐then‐impute demonstrating lower bias than the impute‐then‐exclude strategies for the highest level of missingness investigated (60%).

Estimates of the ACE(t) for the complete case analysis had the most variability, with similar estimates of coverage and standard errors for the MI strategies (Table 2). The impute‐then‐exclude strategies outperformed exclude‐then‐impute more notably in other simulation settings where stronger correlation existed between covariates; for further details, see the Supplement.

### 3.2 Plasmode Simulations

To complement the parametric simulations, we also compared MI strategies using plasmode simulations. Under the plasmode approach, we resampled observations from a real data set to generate simulated data that preserved the empirical relationships among covariates while generating simulated outcome data according to a known treatment‐outcome relationship [34]. The plasmode simulation approach allowed us to assess the performance of MI methods for handling missingness in eligibility criteria in data reflecting the real‐world data distribution of the de‐identified data set. The plasmode simulations also allowed us to specify a known causal effect and missingness mechanism.

To generate simulated data sets, we first estimated the outcome and censoring processes observed in the original data containing no missingness using an adjusted Cox proportional hazards model. For the plasmode simulations, our outcome of interest was progression or all‐cause death, whichever occurred earlier. We modeled the time from the start date of first‐line therapy to the outcome. Individuals who remained alive at the data cut‐off date of November 1, 2023, were censored. We were interested in two first‐line therapy combinations: Letrozole and Palbociclib versus Fulvestrant and Palbociclib. We considered a number of relevant covariates that were also considered in a previous trial emulation [20] for comparing treatments of advanced breast cancer: Age (categorical), ECOG PS, race/ethnicity (Black, Latinx, White, other), time since initial diagnosis, visceral disease (binary, whether cancer spread to visceral tissue such as adrenal, liver, lung, peritoneum, pleura, ovary, or pancreas), non‐visceral disease (binary, whether cancer spread to non‐visceral tissue such as bone, distant lymph node, spleen, or skin), whether the case represented disease progression or de novo metastatic diagnosis, disease‐free interval (categorical), number of metastatic sites (categorical), prior use of osteoporosis medications (binary), osteoporosis history (binary), prior use of antihypertensive medications (binary), anemia history (binary), cardiovascular disease history (binary), body mass index (BMI), and geographic region in the United States (categorical). ECOG PS greater than 2 was considered to be an exclusion criterion, subject to missingness.

We then simulated outcome data using a model that included an interaction between treatment and whether ECOG PS was greater than 2, fixing the treatment effect coefficient to be log(2.0). The censoring model and outcome model were used to generate subject‐specific Breslow estimates of the probability of progression‐or‐death‐free and censoring‐free survival in such a way that preserved the event rate observed in the original data [35, 36]. The coefficients used for the outcome and censoring models can be viewed in the Supplement.

We then generated data sets of size n=1400, which was approximately the size of the complete case sample in the breast cancer cohort. For the simulated data sets, we sampled covariate vectors with replacement from the observed complete case data. For simulating outcomes, we used the sampled covariate data, the specified outcome model described above, and censored based on our estimated censoring model. We also increased the confounding effect on the outcome by ECOG PS, a variable used for assessing eligibility that was also subject to notable amounts of missingness in the real‐world data. ECOG PS describes a patient's overall functional status. ECOG PS scores range from 0 to 5, where higher scores reflect decreased levels of functioning [37]. In simulations and analyses, we categorized ECOG scores as 0, 1, 2, or >2. To better illustrate how the performance of alternative methods differs under increased confounding by an eligibility‐defining variable, we increased the magnitude of coefficients for ECOG = 1 and ECOG = 2. The estimated coefficients for ECOG = 1 and ECOG = 2 in the complete case data were about log(1.19) and log(1.36), respectively; for the plasmode simulations, these coefficient values were set to log(1.67) and log(2.50). All coefficient values used for simulating outcomes in the plasmode simulations can be found in the Supplement.

After simulating the complete data set, we simulated missing at random (MAR) missingness in ECOG PS. In a previous trial emulation from the literature, which also noted missingness in ECOG PS, the authors justified the missing at random assumption since missingness in the data occurred at least partly due to changes in reporting standards among oncology practices over time [20]. Here, we proceeded with assuming that missingness was MAR in ECOG PS, and simulated that missingness in the plasmode data sets. In the full data set, there was about 30% missingness in ECOG PS values overall. To simulate missingness for these plasmode simulations, we defined a missingness model based on other covariates, an event indicator for cancer progression or death, and the cumulative hazards divided by its standard deviation ($H^{*}$), while targeting a fixed proportion of missingness. Similar to the missingness model used for the parametric simulations, the missingness model was:

$$logit(P(R_{Z}=0))=\alpha +\beta_{X}\text{X}+\beta_{\delta }\delta +\beta_{A}A+\beta_{H^{*}}H^{*},$$

where Z represents the ECOG score for the plasmode simulations, and X includes all covariates included in the outcome model except for ECOG. We set the coefficients for missingness for BMI and number of metastatic sites to be 0.1, while the coefficient of missingness for the remaining covariates was set to 1. We also had $\beta_{\delta }=\beta_{H^{*}}=1$ and $\beta_{A}=2$. As in other models, α was chosen to target a set proportion of missingness. We considered missingness proportions ranging from 10% to 50% for ECOG PS.

Also, similar to the parametric simulations, we simulated a large super‐population for estimating the unconditional ACE(t). We estimated the ACE(t) at the median time to survival for the super‐population and imputed missingness in ECOG PS with MICE. Across the 1000 simulation replicates, we obtained estimates of the ACE and the percent relative bias of the ACE with accompanying estimates of RMSE and MCE. Bootstrap confidence intervals were also used to estimate coverage probabilities.

**FIGURE 2:**
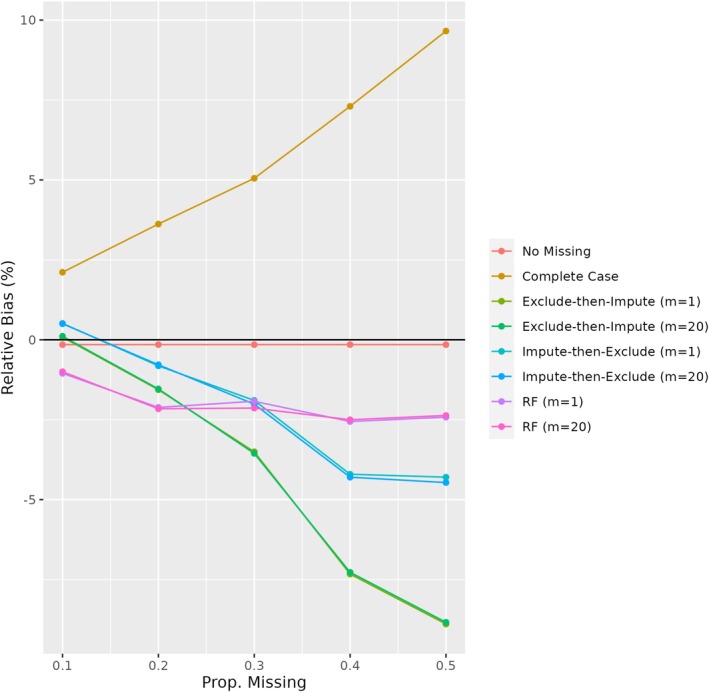
Relative bias of missing data strategies for proportion missingness in ECOG performance status ranging from 0.1 to 0.5 based on plasmode simulations. Results are based on 1000 simulation iterations with sample sizes of n=1400. RF = random forest.

As seen in Figure 2, the pattern of relative bias observed in plasmode simulations was similar to that observed in the parametric simulations. Complete case analysis had the most bias, especially for proportions of missingness in ECOG performance status greater than 30%. We also observed that the performance of exclude‐then‐impute was poorer than other MI‐based approaches, with more bias than other approaches for levels of missingness in ECOG performance status greater than 30%. Impute‐then‐exclude approaches, including those using random forest, tended to result in minimal bias. The MI strategies yielded approximately nominal coverage, with greater variability in estimates of the ACE observed under the exclude‐then‐impute approach (Table 3).

**TABLE 3:** Plasmode simulation results for relative bias, 95% confidence interval coverage probabilities, RMSE, and MCE for relative bias of estimates of ACE. Results are reported for different methods under 30% missingness in ECOG PS.

Method	Relative bias (%)	Coverage	RMSE	MCE for relative bias
No missing data	−0.25	95.4	0.022	0.330
Complete case	2.38	94.0	0.026	0.383
Exclude‐then‐Impute parametric	−1.55	94.7	0.023	0.349
Impute‐then‐Exclude parametric	−1.27	95.6	0.022	0.327
Impute‐then‐Exclude random forest	−0.83	95.8	0.021	0.309

## 4 Real Data Analysis

Using the missing data strategies described above, we conducted a target trial emulation comparing Fulvestrant/Palbociclib to Letrozole/Palbociclib in the real‐world cohort described in Section 2. The sample size was 3825 prior to applying exclusion criteria; 1310 individuals initiated treatment with Fulvestrant/Palbociclib and 2515 with Letrozole/Palbociclib. A comparison of patient demographic and clinical characteristics, including missingness in these variables, can be found in Table 4. Most variables were similar between the two treatment groups. There was a higher proportion of individuals who were not newly diagnosed (90.2%) or who did not take osteoporosis medications (92.9%) in the Fulvestrant/Palbociclib group compared to Letrozole/Palbociclib (61.2%, 79.8%, respectively). The year of treatment start also differed between the two treatment groups.

**TABLE 4:** Patient characteristics for oncology EHR‐derived metastatic breast cancer cohort, overall and stratified by treatment arm.

	Total (N=3825)	Fulvestrant/Palbociclib (N=1310)	Letrozole/Palbociclib (N=2515)
Age			
Mean (SD)	65.9 (10.3)	66.6 (10.1)	65.6 (10.4)
Age, N (%)			
25–44	88 (2.3%)	18 (1.4%)	70 (2.8%)
44–54	425 (11.1%)	148 (11.3%)	277 (11.0%)
55–64	1175 (30.7%)	385 (29.4%)	790 (31.4%)
65–74	1268 (33.2%)	431 (32.9%)	837 (33.3%)
75–84	831 (21.7%)	312 (23.8%)	519 (20.6%)
85+	37 (1.0%)	16 (1.2%)	21 (0.8%)
Missing	1 (0.0%)	0 (0%)	1 (0.0%)
Gender, N (%)			
Female	3802 (99.4%)	1299 (99.2%)	2503 (99.5%)
Male	23 (0.6%)	11 (0.8%)	12 (0.5%)
Race/Ethnicity, N (%)			
Black	345 (9.0%)	120 (9.2%)	225 (8.9%)
Latinx	235 (6.1%)	94 (7.2%)	141 (5.6%)
Other	616 (16.1%)	189 (14.4%)	427 (17.0%)
White	2629 (68.7%)	907 (69.2%)	1722 (68.5%)
ECOG Performance Status, N (%)			
0	1230 (32.2%)	454 (34.7%)	776 (30.9%)
1	1001 (26.2%)	367 (28.0%)	634 (25.2%)
2	321 (8.4%)	133 (10.2%)	188 (7.5%)
> 2	99 (2.6%)	43 (3.3%)	56 (2.2%)
Missing	1174 (30.7%)	313 (23.9%)	861 (34.2%)
ER‐Negative, N (%)			
No	227 (5.9%)	102 (7.8%)	125 (5.0%)
Yes	3583 (93.7%)	1207 (92.1%)	2376 (94.5%)
Missing	15 (0.4%)	1 (0.1%)	14 (0.6%)
HER‐2 Positive, N (%)			
No	3708 (96.9%)	1276 (97.4%)	2432 (96.7%)
Yes	71 (1.9%)	26 (2.0%)	45 (1.8%)
Missing	46 (1.2%)	8 (0.6%)	38 (1.5%)
CNS Metastases, N (%)			
No	3785 (99.0%)	1297 (99.0%)	2488 (98.9%)
Yes	30 (0.8%)	11 (0.8%)	19 (0.8%)
Missing	10 (0.3%)	2 (0.2%)	8 (0.3%)
Letrozole or Anastrozole ≤ 12 Months from Index Date, N (%)			
No	3600 (94.1%)	1256 (95.9%)	2344 (93.2%)
Yes	225 (5.9%)	54 (4.1%)	171 (6.8%)
CDK 4/6 inhibitor ≤12 Months from Index Date, N (%)			
No	3752 (98.1%)	1300 (99.2%)	2452 (97.5%)
Yes	73 (1.9%)	10 (0.8%)	63 (2.5%)
Time Since Initial Diagnosis (years)			
Mean (SD)	5.99 (6.57)	6.51 (5.50)	5.73 (7.05)
Missing	8 (0.2%)	2 (0.2%)	6 (0.2%)
BMI			
Mean (SD)	29.0 (6.69)	28.9 (6.68)	29.0 (6.69)
Missing	304 (7.9%)	38 (2.9%)	266 (10.6%)
Visceral Disease, N (%)			
No	3449 (90.2%)	1179 (90.0%)	2270 (90.3%)
Yes	366 (9.6%)	129 (9.8%)	237 (9.4%)
Missing	10 (0.3%)	2 (0.2%)	8 (0.3%)
Number of Metastatic Sites, N (%)			
1	1176 (30.7%)	360 (27.5%)	816 (32.4%)
2	1032 (27.0%)	334 (25.5%)	698 (27.8%)
3	733 (19.2%)	273 (20.8%)	460 (18.3%)
4+	874 (22.8%)	341 (26.0%)	533 (21.2%)
Missing	10 (0.3%)	2 (0.2%)	8 (0.3%)
US Geographic Region, N (%)			
Midwest	480 (12.5%)	194 (14.8%)	286 (11.4%)
Northeast	480 (12.5%)	179 (13.7%)	301 (12.0%)
South	1283 (33.5%)	438 (33.4%)	845 (33.6%)
West	521 (13.6%)	191 (14.6%)	330 (13.1%)
Missing	1061 (27.7%)	308 (23.5%)	753 (29.9%)
Metastatic Disease at Diagnosis, N (%)			
No	1096 (28.7%)	126 (9.6%)	970 (38.6%)
Yes	2721 (71.1%)	1182 (90.2%)	1539 (61.2%)
Missing	8 (0.2%)	2 (0.2%)	6 (0.2%)
Disease‐free Interval^a^			
≤12 months	2442 (63.8%)	861 (65.7%)	1581 (62.9%)
> 12 months	1383 (36.2%)	449 (34.3%)	934 (37.1%)
Osteoporosis Medications, N (%)			
No	3224 (84.3%)	1217 (92.9%)	2007 (79.8%)
Yes	239 (6.2%)	76 (5.8%)	163 (6.5%)
Missing	362 (9.5%)	17 (1.3%)	345 (13.7%)
History of Anemia, N (%)			
No	3593 (93.9%)	1201 (91.7%)	2392 (95.1%)
Yes	232 (6.1%)	109 (8.3%)	123 (4.9%)
History of Osteoporosis, N (%)			
No	3501 (91.5%)	1142 (87.2%)	2359 (93.8%)
Yes	324 (8.5%)	168 (12.8%)	156 (6.2%)
Cardiovascular Disease History, N (%)			
No	3651 (95.5%)	1238 (94.5%)	2413 (95.9%)
Yes	174 (4.5%)	72 (5.5%)	102 (4.1%)
Treatment Start Year, N (%)			
2014–2015	309 (8.1%)	35 (2.7%)	274 (10.9%)
2016	524 (13.7%)	154 (11.8%)	370 (14.7%)
2017	578 (15.1%)	206 (15.7%)	372 (14.8%)
2018	642 (16.8%)	235 (17.9%)	407 (16.2%)
2019	596 (15.6%)	222 (16.9%)	374 (14.9%)
2020	582 (15.2%)	243 (18.5%)	339 (13.5%)
2021	594 (15.5%)	215 (16.4%)	379 (15.1%)

^a^ Disease‐free interval was the time between the last treatment prior to the first record of metastasis and the date of initial metastasis. If no treatment was present prior to metastasis, then the individual was classified as “newly metastatic.”

The amount of missingness in ECOG PS was about 31%. Other variables with more than 5% missingness were BMI (8% missing), osteoporosis medication usage (10%), and US geographic region (28% missing). For all variables subject to missingness, we used multiple imputation assuming MAR missingness. However, due to collinearity among history of anemia, history of osteoporosis, history of cardiovascular disease, and osteoporosis medication usage, history of anemia, history of osteoporosis, history of cardiovascular disease, and osteoporosis medication usage, these variables were dropped from the imputation model and subsequent analyses.

We used eligibility criteria described in Section 3.2, based on eligibility criteria used in a previously conducted randomized trial [19] and trial emulation [20]. In the randomized trial, the primary outcome was progression‐free survival, while in the previous target trial emulation, the outcome was all‐cause mortality. Both of these studies examined a 3 year time‐frame, so we chose to estimate the unconditional ACE at 3 years while considering and reporting results for both outcomes.

**FIGURE 3:**
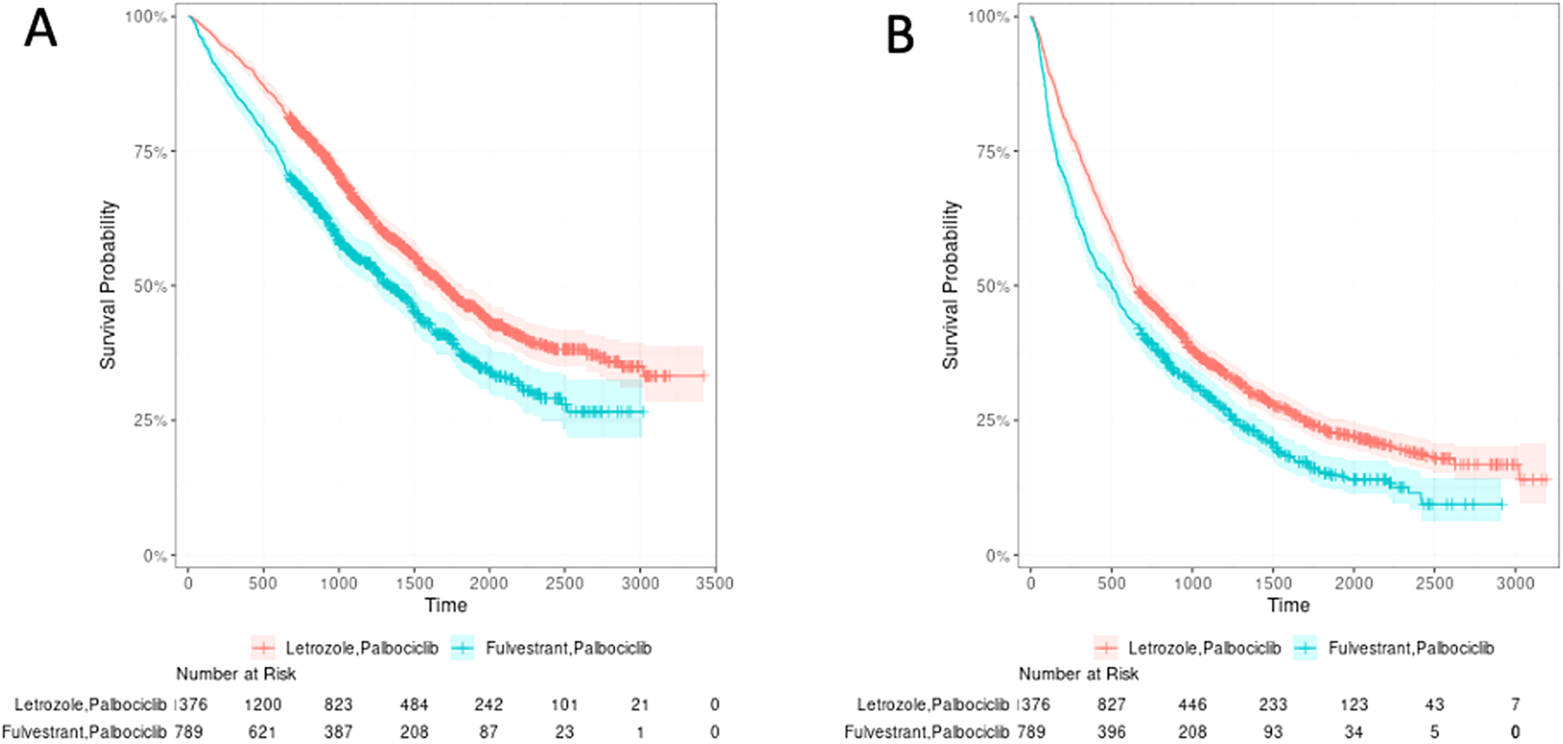
Kaplan–Meier estimates for overall survival (A) and progression‐free survival (B) by treatment group for patients with metastatic breast cancer included in the target trial emulation.

First focusing on results for progression‐free survival, unadjusted 3‐year progression‐free survival was 35.7% (95% CI: 33.2–38.4) in the Letrozole/Palbociclib group and 29.6% (95% CI: 26.5–33.1) in the Fulvestrant/Palbociclib group (Figure 3B). These unadjusted estimates of survival were obtained in the complete case sample. In comparison, the PARSIFAL trial [19] observed about 40% progression‐free survival at 3‐years in both treatment groups, with a slightly higher progression‐free survival rate in the Letrozole/Palbociclib group. Estimates of the ACE and accompanying 95% confidence intervals at three years are shown in Table 5. Results of all approaches were similar, although the complete case analysis was somewhat less efficient than the MI approaches. Under the exclude‐then‐impute approach, the estimate of the ACE was further from 0 than the other approaches, −0.055 versus −0.048. As previously mentioned, the PARSIFAL trial [19] found no significant difference in survival between the two treatment combination groups. Estimates of the ACE that were further from 0 suggest more of a non‐null difference in treatment effects, in contrast to the previous clinical trial finding. The result for the exclude‐then‐impute strategy was a greater departure from the randomized trial result, which showed that the two treatment groups did not have statistically significant differences in progression‐free survival.

**TABLE 5:** Estimates of progression‐free survival probabilities for the two treatment combinations at 3 years in the metastatic breast cancer cohort, ACE for difference in progression‐free survival probability, and 95% confidence intervals for the ACE for varying missingness strategies. Individuals treated with Letrozole and Palbociclib appeared to have better progression‐free survival than individuals treated with Fulvestrant and Palbociclib.

	Est. survival under Fulvestrant‐Palbociclib	Est. survival under Letrozole‐Palbociclib	ACE estimate	95% confidence interval
Complete case	0.303	0.329	−0.027	(−0.068, 0.020)
Exclude‐then‐Impute parametric	0.300	0.355	−0.055	(−0.085, −0.025)
Impute‐then‐Exclude parametric	0.288	0.336	−0.048	(−0.077, −0.016)
Impute‐then‐Exclude random forest	0.288	0.336	−0.048	(−0.079, −0.018)

We found that the 3‐year overall survival was 66.5% (95% CI: 63.9–69.1) in the Letrozole/Palbociclib group and 55.7% (95% CI: 52.3–59.4) in the Fulvestrant/Palbociclib group (Figure 3A). In a previous target trial emulation [20], the 3‐year overall survival rates were estimated as 59.5% (95% CI: 55.5–63.7) and 57.75% (95% CI: 49.6–67.1) in the Letrozole/Palbociclib and Fulvestrant/Palbociclib groups, respectively. The PARSIFAL randomized trial [19] noted 3‐year overall survival rates of 77.1% (95% CI: 70.2–82.5) and 79.4% (95% CI: 73.1–84.4) in the Letrozole/Palbociclib and Fulvestrant/Palbociclib groups, respectively. Table 6 displays estimates of the ACE and accompanying 95% confidence intervals for the various missingness strategies. In this case, the three MI strategies led to similar conclusions. As expected, complete case analysis was less efficient than the MI approaches, which all had similar standard errors.

**TABLE 6:** Estimates of overall survival probabilities for the two treatment combinations at 3 years in the metastatic breast cancer cohort, ACE for difference in overall survival probability, and 95% confidence intervals for the ACE for varying missingness strategies. Individuals treated with Letrozole and Palbociclib appeared to have better overall survival than individuals treated with Fulvestrant and Palbociclib.

	Est. survival under Fulvestrant‐Palbociclib	Est. survival under Letrozole‐Palbociclib	ACE estimate	95% confidence interval
Complete case	0.594	0.634	−0.040	(−0.082, 0.006)
Exclude‐then‐Impute parametric	0.605	0.663	−0.058	(−0.086, −0.028)
Impute‐then‐Exclude parametric	0.587	0.647	−0.060	(−0.089, −0.023)
Impute‐then‐Exclude random forest	0.589	0.649	−0.060	(−0.090, −0.026)

## 5 Discussion

Target trial emulation allows researchers to leverage real‐world data, such as EHR, to explore similar goals to randomized clinical trials. Conducting trial emulations can illuminate or supplement previously‐published findings from clinical trials. Target trial emulations could also provide insight into settings where implementing clinical trials may be infeasible. The target trial emulation framework first introduced by Hernan and Robins [4] includes specifying key components of observational study design, including eligibility criteria for inclusion in the study. EHR may be subject to various data quality issues. Consequently, target trial emulation studies using EHR must also take care to address these challenges. Missingness in key variables from EHR data is one issue that may arise. In the target trial emulation framework, there is a dearth of information on how to best address missingness in eligibility criteria. In the case where eligibility‐defining variables are observed at least partly for the people in the population, our work helps fill this gap for the target trial emulation framework, focusing on using multiple imputation strategies for eligibility criteria while estimating causal treatment effects for survival outcomes from EHR data.

Specifically, we compared the performance of MI strategies for MAR missingness in an eligibility‐defining variable for target trial emulation. We focused on estimation of the ACE with survival outcomes and used parametric and plasmode simulations in addition to analyzing real‐world data for advanced breast cancer patients. In our simulations, we found that multiply imputing with exclude‐then‐impute had more bias than impute‐then‐exclude strategies under most scenarios investigated. The performance of exclude‐then‐impute notably declined (i.e., resulted in increased bias) when eligibility criteria with missingness had strong associations with the outcome or other covariates. While the impute then exclude strategies reduced bias, they did not completely eliminate bias, which increased with increasing amounts of missing data. The bias in all investigated approaches arises because the imputed inclusion criterion is also an effect modifier. Any degree of error in the included study population results in contamination of the effect of treatment and, therefore, bias. This result was previously observed by Austin et al. [13]. For the two impute‐then‐exclude strategies, random forests reduced bias relative to traditional parametric MICE. We also note that we assume MAR missingness in the eligibility variable, but did not examine settings in which missingness was MNAR. Had the missing data mechanisms been MNAR, we would expect that all MI strategies would fail to perform well, including the impute‐then‐exclude strategies.

We used bootstrap standard errors because previous studies identified bias in standard errors estimated using Rubin's Rules applied to data in which eligibility criteria have been imputed [12, 13]. This bias arises due to incompatibility between imputation and outcome models for the impute‐then‐exclude setting [15]. While Giganti and Shepherd [15] have explored an alternative variance estimator from Robins and Wang [38] that uses components derived from imputation and analysis models, this estimator is challenging to implement and is not implemented in standard software. Bootstrap estimates performed well and were computationally feasible.

The previous randomized trial and trial emulation comparing Fulvestrant/Palbociclib to Letrozole/Palbociclib found no difference in effectiveness between the two treatment combinations for either progression‐free survival or overall survival. When the outcome was progression‐free survival, our estimated 3‐year survival rates were notably lower than those of the previous randomized trial [19]. For overall survival using data in the real‐world database, all MI strategies estimated non‐null treatment effects. One possible explanation for this discrepancy in the results could be inherent differences between the EHR and trial populations. Clinical trial populations tend to be healthier overall, which could lead to better outcomes in the clinical trial populations than in the EHR. We noted that our estimated 3‐year overall survival rates in the two treatment groups were higher than those from the previous trial emulation [20] but lower than those reported in the PARSIFAL trial [19]. In studying individuals with poorer outcomes, treatment effects may have been more pronounced, which might help explain the estimated non‐null treatment effects.

As an empirical study, our work is limited by the range of simulation scenarios investigated. Performance of alternative methods in settings beyond those investigated here may differ from the results we observed. Future work could expand on our studies by introducing more complicated relationships among variables, which could also exist within EHR data. These additional explorations could include examining results when simulating greater correlation among certain variables or observing results under more complex missingness patterns for different variables to further assess the benefit in accuracy under impute‐then‐exclude strategies.

Our work leveraged oncology EHR data to investigate strategies that help to facilitate the target trial emulation paradigm in real‐world data that may lack complete information on key variables. Particularly with higher amounts of missingness in eligibility‐defining variables, the ordering of imputation and application of eligibility criteria can produce different treatment effect estimates. We found that impute‐then‐exclude strategies usually resulted in lower bias than complete case analysis and exclude‐then‐impute. This ordering should be taken into account in future target trial emulation studies with eligibility criteria subject to missingness. Importantly, transparent reporting of how missing data have been handled in trial emulation is key to evaluating the validity of results.

## Funding

R.M. reports receiving research grants from Merck and Astellas. KL and RH report receiving grant support from the National Institute of Aging, National Institutes of Health (R21AG075574).

## Conflicts of Interest

R.M. reports receiving research grants from Merck and Astellas; serving as a consultant for Merck, Astellas, BMS, Seagen, and Roche; and serving as an expert witness for King & Spalding.

## Supporting information


**Data S1**: Supporting Information.

## Data Availability

The data that support the findings of this study were originally generated by and are the property of Flatiron Health Inc., which has restrictions prohibiting the authors from making the data set publicly available. Requests for data sharing by license or by permission for the specific purpose of replicating results in this manuscript can be submitted to PublicationsDataAccess@flatiron.com.
