# Supplementary material for: Missingness in Eligibility Criteria for Target Trial Emulation in EHR With Survival Outcomes
Source: Stat Med. 2026 Apr 7;45(8-9):e70500. doi: 10.1002/sim.70500 (PMC13054924; doi:10.1002/sim.70500)
Supplement: Supplementary file 1 — Data S1: Supporting Information. [file SIM-45-0-s001.pdf]

SUPPLEMENT FOR "MISSINGNESS IN ELIGIBILITY CRITERIA FOR TARGET TRIAL EMULATION IN EHR WITH SURVIVAL OUTCOMES"

1 | ADDITIONAL PARAMETRIC SIMULATION RESULTS

Modifying the relationships among some of the variables could lead to bigger differences in the performance among MI strategies. One additional simulation we considered was when we strengthened the relationship between X and Z. Specifically, we modified the data generation mechanism such that $X \sim N(-1.6 + 0.8 * Z, 1)$. All other details remained the same. Under this setting, we observed greater than 10% relative bias for the exclude-then-impute approach while the impute-then-exclude approaches led to less than 5% relative bias. Estimates of the ACE from the impute-then-exclude strategies also had less variability than those from complete case or exclude-then-impute. In this simulation setting, we also observed approximately nominal confidence interval coverage for the impute-then-exclude approaches but see notable under-coverage for the exclude-then-impute approach. Results are shown in Table 1 and Figure 1.

Another consideration could be the performance of the MI strategies with a larger sample size. In the main paper, samples of size $n = 1,000$ were randomly sampled from the same super-population for 1,000 Monte Carlo simulation iterations per simulation scenario. To explore results with a larger sample size, we randomly sampled samples of size $n = 10,000$ from the same super-population following the same approach described in the main text. Results are shown in Table 2 and Figure 2. Similar to what we noted in the main text when randomly drawing smaller samples of size $n = 1,000$, the complete case analysis was highly biased with increasing amounts of bias with increasing amounts of missingness in the eligibility criteria. Among the MI strategies, exclude-then-impute once again performed more poorly than the impute-then-exclude strategies for the majority of scenarios investigated. However, at the highest levels of missing data exclude-then-impute demonstrated lower bias than the impute-then-exclude strategies.

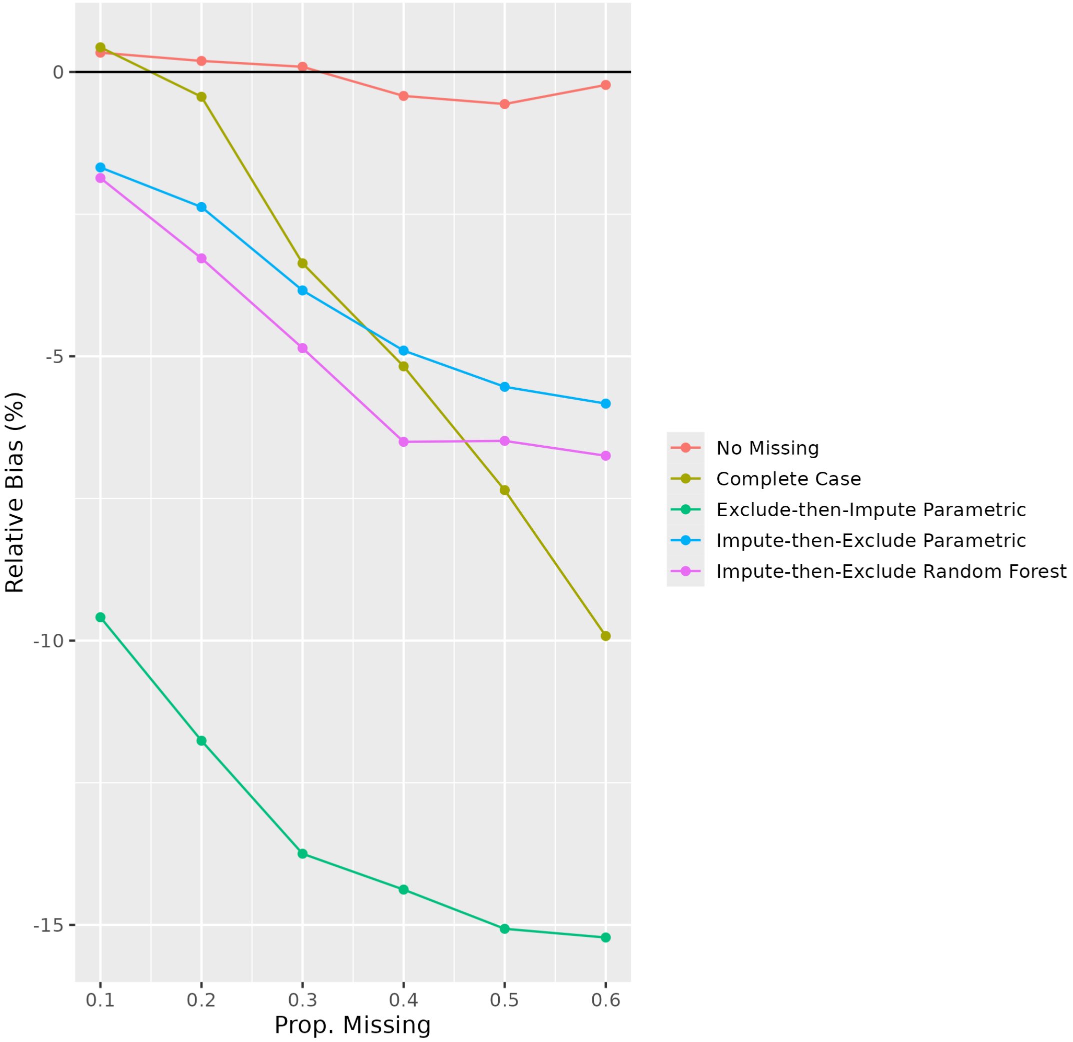

FIGURE 1 Relative bias of missing data strategies for proportion missingness in an eligibility-defining variable ranging from 0.1 to 0.6 based on parametric simulations. Results are based on 1,000 simulation iterations with sample sizes of $n = 1000$.

TABLE 1 Simulation results for relative bias, 95% confidence interval coverage probabilities, RMSE, and MCE for relative bias of estimates of ACE. Results are reported for different methods under 30% missingness in Z.

Method	Relative Bias (%)	Coverage	RMSE	MCE for Relative Bias
No missing data	0.22	95.1	0.019	0.410
Complete Case	-3.23	93.5	0.025	0.531
Exclude-then-Impute Parametric	-13.74	75.9	0.026	0.347
Impute-then-Exclude Parametric	-3.93	94.1	0.020	0.419
Impute-then-Exclude Random Forest	-4.83	95.6	0.020	0.407

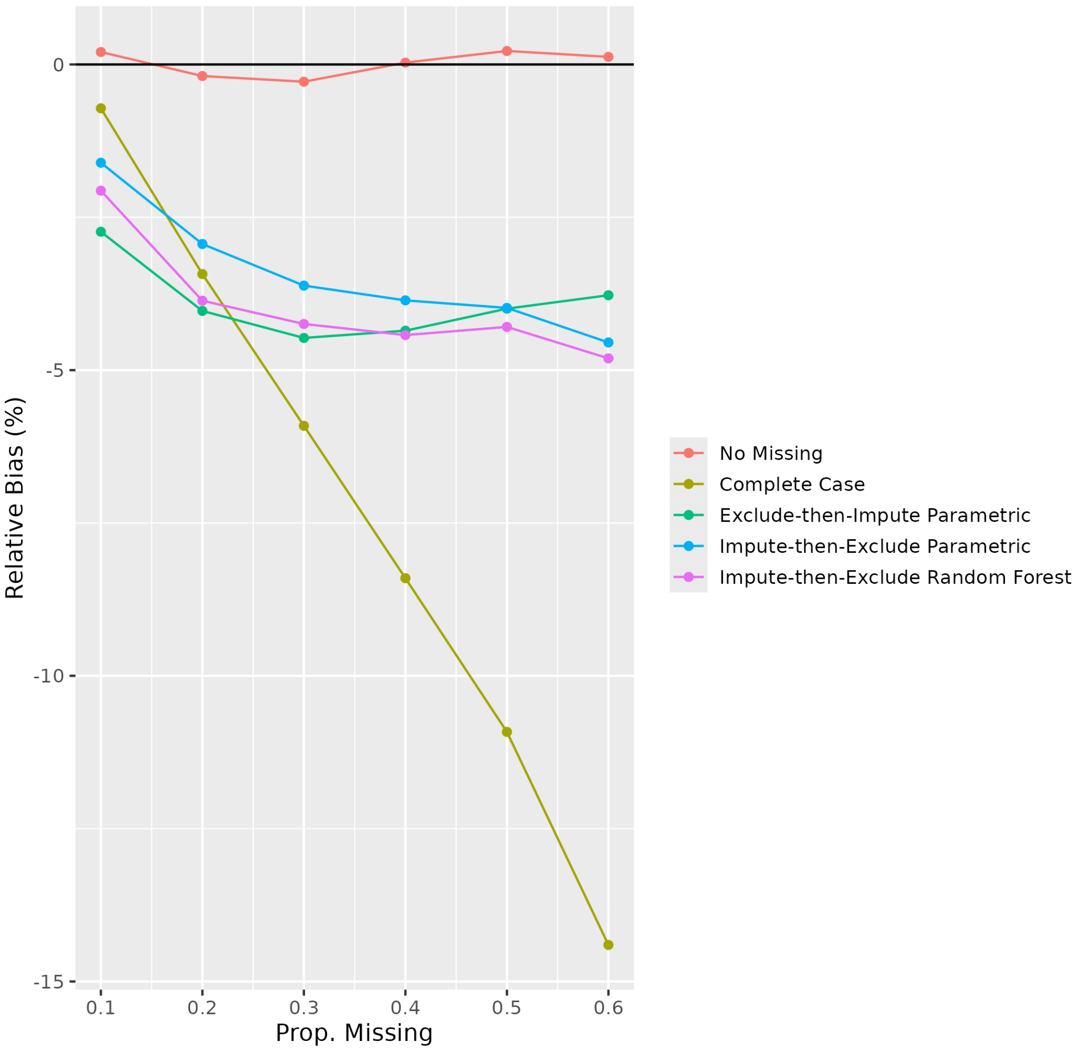

FIGURE 2 Relative bias of missing data strategies for proportion missingness in an eligibility-defining variable ranging from 0.1 to 0.6 based on parametric simulations. Results are based on 1,000 simulation iterations with sample sizes of $n = 10,000$.

TABLE 2 Simulation results for relative bias, 95% confidence interval coverage probabilities, root mean squared error (RMSE), and Monte Carlo error (MCE) for relative bias of estimates of the average causal effect (ACE). Results are reported for different methods under 30% missingness in Z.

Method	Relative Bias (%)	Coverage	RMSE	MCE for Relative Bias
No missing data	-0.26	96.1	0.006	0.122
Complete Case	-5.90	79.6	0.012	0.161
Exclude-then-Impute Parametric	-4.45	81.4	0.009	0.115
Impute-then-Exclude Parametric	-3.55	87.9	0.008	0.126
Impute-then-Exclude Random Forest	-3.90	97.5	0.010	0.155

2 | ADDITIONAL PLASMODE SIMULATION DETAILS

For simulating data for the plasmode simulations, coefficients (on the hazard scale) estimated from the complete case data for the outcome and censoring models are provided in Table 3 and Table 4, respectively.

TABLE 3 Estimated coefficients on the hazard scale from the complete case data for the outcome model used in plasmode simulations.

Variable	Estimate	Standard Error	Statistic	P-Value	Lower Confidence Limit	Upper Confidence Limit
Fulvestrant,Palb.	1.100	0.066	1.448	0.148	0.967	1.252
ECOG>2	1.264	0.259	0.903	0.366	0.760	2.101
ECOG=1	1.187	0.067	2.553	0.011	1.041	1.354
ECOG=2	1.358	0.098	3.118	0.002	1.120	1.646
ECOG>2 * Fulvestrant, Palbociclib	1.047	0.351	0.131	0.896	0.527	2.081
Age (44-54)	0.966	0.256	-0.134	0.893	0.585	1.596
Age (55-64)	1.201	0.249	0.737	0.461	0.737	1.956
Age (65-74)	0.873	0.249	-0.546	0.585	0.535	1.422
Age (75-84)	1.194	0.253	0.701	0.483	0.727	1.962
Age (85+)	2.050	0.369	1.945	0.052	0.994	4.224
Ethnicity (Black)	1.210	0.106	1.788	0.074	0.982	1.490
Ethnicity (Latinx)	1.076	0.129	0.570	0.569	0.835	1.387
Ethnicity (Other)	1.411	0.119	2.899	0.004	1.118	1.781

Time Since Initial Diagnosis (Years)	0.959	0.007	-6.461	0.000	0.946	0.971
BMI	0.989	0.005	-2.285	0.022	0.980	0.998
Visceral Disease (Yes)	0.893	0.228	-0.493	0.622	0.571	1.398
Non-Visceral Disease (Yes)	0.761	0.203	-1.346	0.178	0.512	1.132
Number of Metastatic Sites (2)	1.913	0.086	7.578	0.000	1.618	2.263
Number of Metastatic Sites (3)	2.468	0.093	9.745	0.000	2.058	2.960
Number of Metastatic Sites (4+)	3.060	0.087	12.912	0.000	2.582	3.627
Region (Northeast)	0.988	0.098	-0.125	0.900	0.815	1.197
Region (South)	0.943	0.081	-0.727	0.467	0.805	1.105
Region (West)	0.926	0.108	-0.710	0.477	0.749	1.145
Newly Diagnosed (Yes)	0.613	0.093	-5.278	0.000	0.511	0.735
Disease-free Interval (>12 months)	0.997	0.063	-0.052	0.959	0.881	1.128

Osteoporosis Medications (Yes)	1.158	0.100	1.472	0.141	0.952	1.409
History of Anemia (Yes)	1.306	0.114	2.338	0.019	1.044	1.635
History of Osteoporosis (Yes)	0.998	0.118	-0.019	0.985	0.791	1.258
Cardiovascular Disease History (Yes)	1.058	0.158	0.357	0.721	0.777	1.442

TABLE 4 Coefficients on the hazard scale from the complete case data for the censoring model used in plasmode simulations.

Variable	Estimate	Standard Error	Statistic	P-Value	Lower Confidence Limit	Upper Confidence Limit
Fulvestrant, Palbociclib.	1.215	0.135	1.440	0.150	0.932	1.583
ECOG>2	1.868	0.524	1.193	0.233	0.669	5.216
ECOG=1	0.996	0.134	-0.027	0.979	0.766	1.296
ECOG=2	1.208	0.207	0.915	0.360	0.806	1.813
ECOG>2 * Fulvestrant, Palbociclib	0.673	0.734	-0.538	0.590	0.160	2.841
Age (44-54)	0.848	0.752	-0.220	0.826	0.194	3.698
Age (55-64)	1.326	0.742	0.381	0.703	0.310	5.674
Age (65-74)	1.452	0.737	0.506	0.613	0.342	6.158
Age (75-84)	1.243	0.746	0.292	0.770	0.288	5.365
Age (85+)	7.008	1.034	1.882	0.060	0.923	53.223

Ethnicity (Black)	1.399	0.236	1.421	0.155	0.880	2.224
Ethnicity (Latinx)	1.257	0.221	1.032	0.302	0.814	1.939
Ethnicity (Other)	1.281	0.297	0.834	0.404	0.716	2.295
Time Since Initial Diagnosis (Years)	0.987	0.011	-1.104	0.270	0.965	1.010
BMI	0.984	0.009	-1.785	0.074	0.967	1.002
Visceral Disease (Yes)	0.980	0.523	-0.038	0.970	0.352	2.734
Non-Visceral Disease (Yes)	1.317	0.482	0.571	0.568	0.512	3.385
Number of Metastatic Sites (2)	1.038	0.147	0.254	0.799	0.779	1.384
Number of Metastatic Sites (3)	0.803	0.205	-1.069	0.285	0.538	1.200
Number of Metastatic Sites (4+)	0.813	0.221	-0.936	0.349	0.527	1.254
Region (Northeast)	1.164	0.194	0.783	0.433	0.796	1.704
Region (South)	1.236	0.162	1.311	0.190	0.900	1.698
Region (West)	1.242	0.219	0.988	0.323	0.808	1.907

Newly Diagnosed (Yes)	0.688	0.187	-2.002	0.045	0.477	0.992
Disease-free Interval (>12 months)	1.199	0.127	1.426	0.154	0.934	1.539
Osteoporosis Medications (Yes)	0.780	0.241	-1.034	0.301	0.487	1.250
History of Anemia (Yes)	1.533	0.250	1.708	0.088	0.939	2.502
History of Osteoporosis (Yes)	1.291	0.232	1.100	0.271	0.819	2.035
Cardiovascular Disease History (Yes)	1.466	0.258	1.485	0.138	0.885	2.430

The true coefficients (on the hazard scale) used for simulating outcomes in the plasmode simulations are shown in Table 3.

TABLE 5 True coefficients on the hazard scale used for simulating outcomes in the plasmode simulations.

**Variable**	**Estimate**
Fulvestrant,Palb.	2.000
ECOG>2	1.264
ECOG=1	1.672
ECOG=2	2.503
ECOG>2 * Fulvestrant, Palbociclib	1.047
Age (44-54)	0.966
Age (55-64)	1.201
Age (65-74)	0.873

Age (75-84)	1.194
Age (85+)	2.050
Ethnicity (Black)	1.210
Ethnicity (Latinx)	1.076
Ethnicity (Other)	1.411
Time Since Initial Diagnosis (Years)	0.959
BMI	0.989
Visceral Disease (Yes)	0.893
Non-Visceral Disease (Yes)	0.761
Number of Metastatic Sites (2)	1.913
Number of Metastatic Sites (3)	2.468
Number of Metastatic Sites (4+)	3.060
Region (Northeast)	0.988
Region (South)	0.943
Region (West)	0.926
Newly Diagnosed (Yes)	0.613
Disease-free Interval (>12 months)	0.997
Osteoporosis Medications (Yes)	1.158
History of Anemia (Yes)	1.306
History of Osteoporosis (Yes)	0.998
Cardiovascular Disease History (Yes)	1.058
